# Aerobic and anaerobic reduction of birnessite by a novel *Dietzia* strain

**DOI:** 10.1186/s12932-015-0026-0

**Published:** 2015-08-08

**Authors:** Huiqin Zhang, Yan Li, Xin Wang, Anhuai Lu, Hongrui Ding, Cuiping Zeng, Xiao Wang, Xiaolei Wu, Yong Nie, Changqiu Wang

**Affiliations:** The Key Laboratory of Orogenic Belts and Crustal Evolution, School of Earth and Space Sciences, Peking University, Beijing, 100871 People’s Republic of China; Department of Energy and Resources Engineering, College of Engineering, Peking University, Beijing, 100871 People’s Republic of China

## Abstract

**Background:**

Mn oxides occur in a wide variety of geological settings and exert considerable influences on the components and chemical behaviors of sediments and soils. Microbial reduction of Mn oxides is an important process found in many different environments including marine and freshwater sediments, lakes, anoxic basins, as well as oxic-anoxic transition zone of ocean. Although the pathway of Mn anaerobic reduction by two model bacteria, *Geobacter* and *Shewanella*, has been intensively studied, Mn bio-reduction is still the least well-explored process in nature. Particularly, reduction of Mn oxides by other bacteria and in the presence of O_2_ has been fewly reported in recent publishes.

**Results:**

A series of experiments were conducted to understand the capability of *Dietzia* DQ12-45-1b in bioreduction of birnessite. In anaerobic systems, Mn reduction rate reached as high as 93% within 4 weeks when inoculated with 1.0 × 10^10^ cells/mL *Dietzia* DQ12-45-1b strains. Addition of AQDS enhanced Mn reduction rate from 53 to 91%. The anaerobic reduction of Mn was not coupled by any increase in bacterial protein concentration, and the reduction rate in the stable stage of day 2–14 was found to be in good proportion to the protein concentration. The anaerobic reduction of birnessite released Mn(II) either into the medium or adsorbed on the mineral or bacteria surface and resulted in the dissolution of birnessite as indicated by XRD, SEM and XANES. Under aerobic condition, the reduction rate was only 37% with a cell concentration of 1.0 × 10^10^ cells/mL, much lower than that in parallel anaerobic treatment. Bacterial growth under aerobic condition was indicated by time-course increase of protein and pH. In contrast to anaerobic experiments, addition of AQDS decreased Mn reduction rate from 25 to 6%. The reduced Mn(II) combined with carbon dioxide produced by acetate metabolism, as well as an alkaline pH environment given by cell growth, finally resulted in the formation of Mn(II)-bearing carbonate (kutnohorite), which was verified by XRD and XANES results. The system with the highest cell concentration of 1.0 × 10^10^ cells/mL gave rise to the most amount of kutnohorite, while concentration of Mn(II) produced with cell concentration of 6.2 × 10^8^ cells/mL was too low to thermodynamically favor the formation of kutnohorite but result in the formation of aragonite instead.

**Conclusion:**

*Dietzia* DQ12-45-1b was able to anaerobically and aerobically reduce birnessite. The rate and extent of Mn(IV) reduction depend on cell concentration, addition of AQDS or not, and presence of O_2_ or not. Meanwhile, Mn(IV) bioreduction extent and suspension conditions determined the insoluble mineral products.

**Electronic supplementary material:**

The online version of this article (doi:10.1186/s12932-015-0026-0) contains supplementary material, which is available to authorized users.

## Background

Manganese is the 10th most abundant element in the Earth’s crust and second only to iron as the transition metal with alternating redox states [1, 2]. More than 30 kinds of Mn oxide/hydroxide minerals ubiquitously distribute in natural environment [2], which are highly chemically active, and have been recognized as being important in controlling the availability and distribution of many trace metals [2–6]. Mn cycling depends on various environmental conditions, such as pH, Eh and temperature etc., which lead to complicated behaviors of Mn such as dissolution, precipitation and phase transformation [2, 7–11]. Microbially influenced transformations of Mn which have been previously reported to take place in soils, sediments, mine tailings, and marine environments, also play an important role in driving geochemical cyclings of Mn [7–12].

The formation of many naturally occurring Mn oxides is found to be associated with microbial Mn(II) oxidation processes [3, 4, 13–15]. Meanwhile, microorganisms were also found to participate in Mn(IV) oxides reduction processes, either by using Mn(IV) as a sole electron acceptor or excreting organics to reduce Mn(IV) as a detoxification mechanism [16–18]. *Geobacter* sp. and *Shewanella* sp. are two representative species of dissimilatory metal reducing bacteria (DMRB) and have been extensively investigated with respect to their ability to reduce Mn(IV) [10, 16, 19–22]. Reduction of Mn(IV) oxides by other DMRB have been seldom reported in recent publishes.

Recent researches carried out the dissimilatory Mn(IV) reduction under anoxic conditions. In the absence of oxygen, some manganese-reducing organisms may use manganese oxides as electron acceptors [16, 17]. While some laboratory studies observed that the presence of oxygen did not inhibit microbial manganese reduction due to the existence of a manganese-reductase system whose activity was inducible by Mn(II) and unaffected by O_2_ [23, 24]. Although Mn(IV) reduction has been more commonly observed in anaerobic conditions, it may also occur in the presence of oxygen.

Factually, biotic manganese reduction is complicated in natural environments and is found to be influenced by various factors. Besides the types of microbial species and O_2_ level, electron shuttles, such as humic acid and quinone-containing compounds, also have great influences on microbial Mn(IV) reduction rates [18, 25, 26]. Lovley [25] proved that addition of humic substances or anthraquinone-2,6-disulfonate (AQDS) greatly stimulated the reduction activity of *Geobacter metallireducens*. Ruebush [26] testified that enzymatic reduction of Mn oxides by membrane fractions from *Shewanella oneidensis* MR-1 was accelerated with addition of AQDS.

In this study, a fermentative facultative anaerobe, *Dietzia* strain DQ12-45-1b, which was isolated from a microaerobic condition, was investigated for reduction of a most common Mn(IV) oxide, birnessite. Given previously reported observations, microbial Mn(IV) reduction by DQ12-45-1b were further studied by examining possible constrains of cell densities, O_2_ and electron shuttles (AQDS) on Mn(IV) reduction rates as well as the resulting Mn-bearing mineral products.

## Methods

### Birnessite preparation

Birnessite was synthesized using the method described by McKenzie [27]. A 30 mL concentrated HCl (AR) was added dropwisely with stirring to a boiling solution containing 0.2 mol KMnO_4_ (AR) dissolved in 350–400 mL of water. After all 30 mL HCl added into the KMnO_4_ solution, the reaction was continued under another 30 min boiling. The precipitate was filtered and washed 15 times with deionized water [18 MΩ conductivity (Reference, Merck Milipore, Germany)] to remove K^+^ and Cl^−^ possibly adsorbed on the mineral surface. The resulting precipitate was dried in air at 45°C over night and then stored for further test and experiment.

### Bacteria

*Dietzia* strain DQ12-45-1b was isolated from oil production water in a deep subterranean oil-reservoir of an oilfield in China [28]. This strain was gram-positive, facultatively anaerobic, non-motile with no flagellum and had the ability to degradation of petroleum hydrocarbons [28]. Batch growth experimental data showed DQ12-45-1b was able to grow on any single substrate of succinate, acetate and glucose, while formate, lactate and citrate could not serve as the sole carbon and energy source for bacterial growth. Therefore, we chose a simple organic of acetate as the electron donor in this study.

The strain was prepared for bioreduction experiments after aerobic enrichment cultivation in Luria–Bertani medium (LB medium, 10 g/L of peptone, 5 g/L of yeast extract and 10 g/L of NaCl) under ambient condition. In bioreduction experiments, strain DQ12-45-1b was cultured anaerobically or aerobically in a medium [29] consisting of (per liter): 6.56 g sodium acetate, 1.19 g (NH_4_)_2_SO_4_, 0.1 g MgSO_4_, 0.043 g CaCl_2_, 0.0012 g FeSO_4_, and 20 mM HEPES buffer at 35°C.

### Bioreduction experiments

Birnessite (final concentration = 0.3 mg/mL) was suspended with the culture medium in serum bottles sealed with blue butyl rubber stoppers (for anaerobic) and in flasks (for aerobic) with total volume of 80 mL. In anaerobic experiments, the medium used for anaerobic cultures was made anoxic in serum bottles with O_2_-free N_2_/CO_2_ gasmix (80:20) and sterilized by autoclaving. *Dietzia* DQ12-45-1b cell was enriched from LB medium by centrifugation at 4,024*g*, and then washed with sterilized culture medium. This procedure was repeated for three times to remove LB medium, and then the cell pellet was suspended with sterilized culture medium and injected into serum bottles with a fixed concentration. In selected experiments, 0.1 mM anthraquinone-2,6-disulfonate (AQDS) was supplied as an electron shuttle. AQDS solution and HEPES buffer were sterilized by filtration with 0.22 μm Millipore filter and injected into the medium with syringes. The control group was identical to the experimental bottles except that cells were replaced with an equal amount of the culture medium (sterile control) or inactivated cells (killed control). All treatments were performed in duplicates. All vials were incubated in a constant temperature shaking table at 35°C. Samplings were conducted in certain time interval in glove box (855AC, Plas-Labs, USA). 3 mL of suspensions was taken out for pH, Mn^2+^ concentration and protein concentration tests to evaluate microbial reduction and the changes of biomass. In aerobic experiments, all processes were identical to those in anaerobic experiments except that oxygen removal was not conducted and the sampling processes were conducted in super clean bench.

### Analytical methods

Mn^2+^ concentration (*C*_*l*_) was measured by ICP-OES (Spectroblue, Spectro, Germany) after removing solids by centrifugation of 1 mL cell-mineral suspension. Protein concentrations were measured by Bradford method [30]. 1 mL sample was centrifuged at 9,391*g* and the supernatant was discarded. 0.2 M NaOH was fully mixed with cell pellet, and the mixture was boiled for 12 min to breakdown the cells and release proteins. The alkali treated sample was then centrifuged and the protein fraction in the supernatant was quantified with the Bradford assay using the standard curve established by bovine serum albumin (BSA) as a standard [30].

Mn average oxidation state (AOS) of the suspensions was calculated after measuring the total Mn content, Mn^2+^ content in the medium and Mn(III)/Mn(IV) content, respectively. First, total Mn content (*C*_*total*_) was measured by dissolving 0.5 mL suspension using 2 mL 0.25 M hydroxylamine hydrochloride, then diluted to 10 mL with 2% HNO_3_, and then the Mn concentration was determined by ICP-OES. Aqueous Mn^2+^ content (*C*_*l*_) in the solution was also determined by ICP-OES. The amount of Mn(IV) in the suspension was measured by reaction with the reductive dye, Leucober-belin blue I (LBB) using the standard curve established by KMnO_4_ as a standard [31]. Oxidized LBB is blue and the color intensity is a function of the amount of Mn(III)/Mn(IV) being reduced to Mn(II). The color intensity was measured for optical density at 620 nm using the spectrophotometer. Amount of electron transfer between Mn(III)/Mn(IV) and Mn(II) was noted as N_e_. A concentration of 10 μM KMnO_4_ equaled 50 μM electron transfer. According to the amount of total Mn, Mn^2+^ and transfer electrons, the Mn AOS was calculated as following equations:

1 $$ {\text{AOS}}_{{({\text{Total Mn}})}} = { 2} + {\text{N}}_{e} /{\text{C}}_{total} $$

2 $$ {\text{AOS}}_{{({\text{insoluble Mn}})}} = { 2} + {\text{N}}_{e} /\left( {{\text{C}}_{total} - {\text{ C}}_{l} } \right) $$

where AOS_(Total Mn)_ means AOS of total Mn in the suspensions and AOS_(insoluble Mn)_ means AOS of insoluble Mn including Mn in minerals and adsorbed on bacteria or minerals.

It is supposed that all Mn(III)/Mn(IV) were reduced to Mn(II). And birnessite reduction rate was calculated as following:

3 $$ {\text{Reduction extent }} = \left( {{\text{AOS}}_{\text{({{Total Mn}})}t} - 2} \right)/ \, \left( {{\text{AOS}}_{{\left( {\text{Total Mn}} \right){\it 0}}} - 2} \right) \times 100\% $$

where AOS_(Total Mn)*0*_ means AOS of total Mn at the original time and AOS_(Total Mn)*t*_ means AOS of total Mn after reaction. AOS_(Total Mn)*0*_ was measured as 3.92.

### Mineral characterization

Mineralogical changes after bioreduction were determined by powder X-ray diffraction (XRD). XRD patterns were recorded using X`pert powder diffractometer (PANalytical B.V., the Netherlands) with CuKα radiation (λ = 0.15406 nm). The instrument was operated at a tube voltage of 40 kV and a tube current of 40 mA. Intensities were measured at 2θ = 5°–70° with 0.02° two-theta steps and a count time of 0.3 s per step.

The mineral micro-morphologies were further characterized by scanning electron microscopy (SEM). Suspensions (1 mL) were washed by deionized water to remove medium on the mineral surface. The samples were dispersed on polished silicon wafer and then mounted on an aluminum SEM stub via conductive tapes and coated with gold using a Denton Desk II Gold Sputter Coater for SEM observations. The samples were observed under a FEI Quanta 200F SEM with an X-ray energy dispersive spectroscopy (SEM/EDS). The SEM was operated at an accelerating voltage of 10 or 15 kV.

The X-ray absorption near-edge data (XANES) at the Mn K-edge of the original and bioreduced samples were recorded at room temperature in transmission mode using ion chambers at beam line BL14W1 of the Shanghai Synchrotron Radiation Facility (SSRF), China. The station was operated with a Si (111) double crystal monochromator with a resolution of 1.3 × 10^−4^ eV. Mn K-edge XANES data were collected over the energy range 6,339–6,839 eV in transmission mode. Each powder sample was sandwiched between two pieces of KAPTON tape located on the beam path. During the measurement, the synchrotron was operated at energy of 3.5 GeV and a maximum current of 250 mA. The photon energy was calibrated with the first inflection point of Mn K-edge in Mn metal foil. Data reduction of experimental XANES spectra was carried out using the software ATHANE 1.2.11. Pre-edge background subtraction and XANES normalization were carried out by fitting a linear polynomial to the pre-edge region and a quadratic polynomial to the postedge region of the absorption spectrum.

## Results and discussion

### Anaerobic reduction of birnessite by DQ12-45-1b

**Fig. 1:**
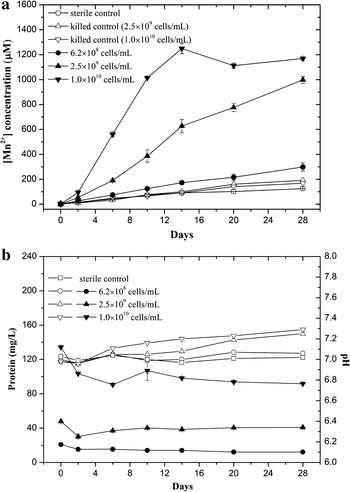
Aqueous Mn^2+^ concentrations (**a**) and protein concentrations (*solid symbol*)/pH values (*hollow symbol*) (**b**) during anaerobic birnessite reduction with different initial cell concentrations.

The results of birnessite reduction by DQ12-45-1b were shown in Fig. 1 and summarized in Table 1. As observed in Fig. 1a, significant amounts of Mn^2+^ were produced in bacterial treatments, which was considerably higher than the concentrations of Mn^2+^ in sterile control and killed control. In the initial 14 days, the amounts of Mn^2+^ increased with time and 6, 21 and 42% of Mn(IV) was reduced with the initial cell concentration of 6.2 × 10^8^, 2.5 × 10^9^ and 1.0 × 10^10^ cells/mL, respectively. By contrast, the chemical reduction extent of Mn(IV) by acetate was below 5%.

**Table 1:** Anaerobic reduction of birnessite under different cell concentrations

Cell concentration (cell/mL)	AOS_(Total Mn)_	AOS_(insoluble Mn)_	Mn^2+^/Mn_(total)_ (%)	Reduction extent (%)
6.2 × 10^8^	3.29	3.44	10	33
2.5 × 10^9^	2.90	3.35	33	53
1.0 × 10^10^	2.13	2.22	39	93
0	3.83	3.91	4	5

All samples were biotreated for 28 days.

Since changes in Mn reduction rate could be roughly estimated from Mn^2+^ release rate, three stages of Mn reduction process could be approximately obtained from Fig. 1a. The first stage is the initial 2 days, when Mn^2+^ concentration increased but with a relatively lower rate than the following several days. Mn^2+^ release rates with initial cell concentrations of 6.2 × 10^8^ and 2.5 × 10^9^ during 2–14 days and that with 1.0 × 10^10^ cells/mL during 2–10 days was calculated to be 12.2, 48.0 and 114.9 μM/day, respectively, showing a positive correlation with the inoculated cell density. However, Mn^2+^ release rates after 14 days decreased to 9.1 and 26.5 μM/day in the treatments with two lower cell concentrations, indicating the bacterial activity associated with Mn bioreduction went down. Even a slight decrease in Mn^2+^ concentration after 14 days was observed with the highest inoculation concentration of 1.0 × 10^10^ cells/mL, suggesting the bioreduction possibly stopped. Besides, tiny amounts of particles were observed in the medium after 14 days. All these evidences indicated the bioreduction of Mn with the highest cell concentration of 1.0 × 10^10^ cells/mL was close to completion at the 14th day. Consistently, the AOS of the residuals were measured to be 2.22 (Table 1) and found to be approaching 2. The slight decrease of Mn^2+^ concentration after 14 days would be explained by adsorption of Mn(II) onto the cell or residual mineral surface, or else by forming Mn(II)/Mn(III) minerals [32–34]. So, in bacterial treatment with cell concentration of 1.0 × 10^10^ cells/mL, almost all Mn(IV) in birnessite was reduced to Mn(II) after 14 days and part of produced Mn(II) was present as insoluble state. There was a positive relationship between the Mn reduction rate and the cell concentration, that the final reduction extent of birnessite was 33, 53 and 93% (Fig. 1; Table 1), corresponding to the cell concentration of 6.2 × 10^8^, 2.5 × 10^9^ and 1.0 × 10^10^ cells/mL, respectively.

Previous studies showed *Shewanella oneidensis* MR-1 could couple its anaerobic growth to Mn(IV) reduction and gain energy from the redox reaction including organics oxidation and metal reduction [16]. Here, acetate served as the electron donor, and birnessite served as the electron acceptor. So, the total reaction could be given as:

4 $$ 3 {\text{MnO}}_{ 2} + {\text{ CH}}_{ 3} {\text{COO}}^{ - } + {\text{ H}}_{ 2} {\text{O}} \mathop{\longrightarrow}\limits 3 {\text{Mn}}^{ 2+ } + {\text{ 2HCO}}_{ 3}^{ - } + {\text{ 3OH}}^{ - } $$

**Fig. 2:**
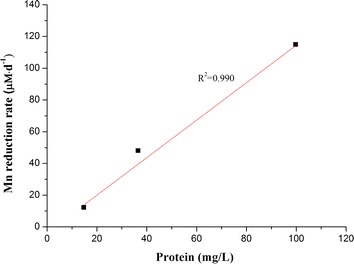
A positive relationship between protein and Mn^2+^ release rate (abscissa values were the mean values of proteins in 2–14 days).

However, the anaerobic reduction of birnessite by DQ12-45-1b were found to be unaccompanied by bacterial growth. In the presence of birnessite as the sole electron acceptor, the concentrations of Mn^2+^ continuously increased in all three bacterial treatments during the stable bioreduction stage (day 2–14), while the bacterial protein concentration kept stable (Fig. 1b). Particularly, the Mn^2+^ release rates were found to be in good proportion to the protein concentrations (Fig. 2). Therefore, we can confirm the anaerobic reduction of birnessite by DQ12-45-1b was not a direct biological process linking Mn reduction with bacterial growth. Considering the results of killed control and the positive correlation between the Mn^2+^ release rates and protein concentrations, we speculate the bioreduction process may be an enzymatic reaction, which needs further demonstration.

### Aerobic reduction of birnessite by DQ12-45-1b

**Fig. 3:**
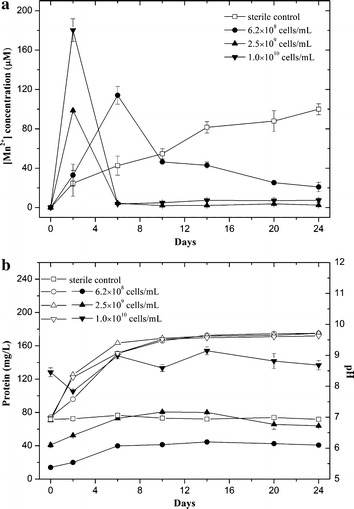
Aqueous Mn^2+^ concentration (**a**) and protein concentration (*solid symbol*)/pH value (*hollow symbols*) (**b**) during aerobic birnessite reduction with different initial cell concentration.

Under aerobic conditions, Mn^2+^ concentration of sterile treatment gradually increased over the experiment (Fig. 3a) due to acetate reduction. In treatments with the initial inoculation cell concentration of 6.2 × 10^8^, 2.5 × 10^9^ and 1.0 × 10^10^ cells/mL, Mn^2+^ concentration in the initial 2 days sharply increased to 33.0, 98.7 and 180.0 μM, respectively. The initial Mn^2+^ release rates also showed a positive correlation with the inoculated cell density. Meanwhile, the pH drastically went up from 6.9 to 8.3 for the two higher cell concentrations even in the presence of HEPES buffer, which lead to the quick precipitation, re-adsorption of Mn(II) on the mineral surface or re-oxidation of Mn(II) as reflected by the abrupt drop of Mn^2+^ concentration to zero in the following days. As expected, some white precipitations were observed in the suspensions with two higher cell concentrations of 2.5 × 10^9^ and 1.0 × 10^10^ cells/mL, indicating the formation of new minerals related to Mn reduction. In the medium with the lowest cell concentration of 6.2 × 10^8^ cells/mL, both the maximum Mn^2+^ generation and the most drastic change in pH were recorded later than in the equivalent treatments with higher cell concentration (approximately at the 6th day). And after the 6th day, the Mn^2+^ concentration did not undergo a sudden change to zero but gradually decreased. No visible precipitates were observed in this treatment.

Bacterial growth under aerobic condition was indicated by time-course increase of protein and pH (Fig. 3b). When grown in pure culture with O_2_ as the terminal electron acceptor, the DQ12-45-1b strains were found to be able to increase the pH value from neutral to alkaline scales (data not shown). Therefore, the rapider increase in both soluble Mn^2+^ and pH values in bacterial treatments than those in sterile control, as well as the obvious positive correlation between the inoculated cell concentration and Mn reduction extent indicated the aerobic Mn reduction was correlated with the bacterial growth. In a separate experimental batch, which was to examine the relationship between the acetate consumption and cell concentration, we observed the depletion of acetate at around the 6th day (Additional file 1: Figure S1). So, along with the depletion of carbon source, the bacterial growth stagnated, and the protein concentration did not increase any more (Fig. 3b). The pH went stable and finally maintained at 9.6–9.7 (Fig. 3b).

After 24 days, the AOS of insoluble Mn in the system with initial cell concentrations of 2.5 × 10^9^ and 1.0 × 10^10^ cells/mL were 3.44 and 3.21, corresponding to 25 and 37% of Mn reduction, respectively (Table 2). The actual Mn reduction extent should be higher than the experimental values, because under alkaline condition, re-oxidation of Mn(II) by O_2_ is feasible [35, 36]. In comparison, the AOS of insolubles with initial cell concentration of 6.2 × 10^8^ cells/mL was 3.92 (Table 2), the same as the original birnessite. Although there were some Mn^2+^ continuously released in the initial 6 days of 6.2 × 10^8^ cells/mL treatment, the Mn^2+^ concentration gradually decreased in the following days (Fig. 3a).

**Table 2:** Aerobic reduction of birnessite under different cell concentrations

Cell concentration (cell/mL)	AOS_(Total Mn)_	AOS_(insoluble Mn)_		Mn^2+^/Mn_(total)_ (%)	Reduction extent (%)
6.2 × 10^8^	3.91	3.92	<1		<1
2.5 × 10^9^	3.44	3.44	<1		25
1.0 × 10^10^	3.21	3.21	<1		37
0	3.88	3.94	3		3

All samples were biotreated for 24 days.

Comparing Mn bioreduction in the presence and absence of O_2_, it could be found that Mn reduction extents under aerobic condition were much lower than those under anaerobic conditions, although stain DQ12-45-1b grew more vigorous under aerobic conditions. These findings suggested O_2_ interfered with birnessite reduction, not only as an alternative electron acceptor to compete with Mn(IV) reduction, but also as an oxidizer leading to re-oxidation of Mn(II) in alkaline pH.

### Effect of AQDS on reduction of birnessite

**Fig. 4:**
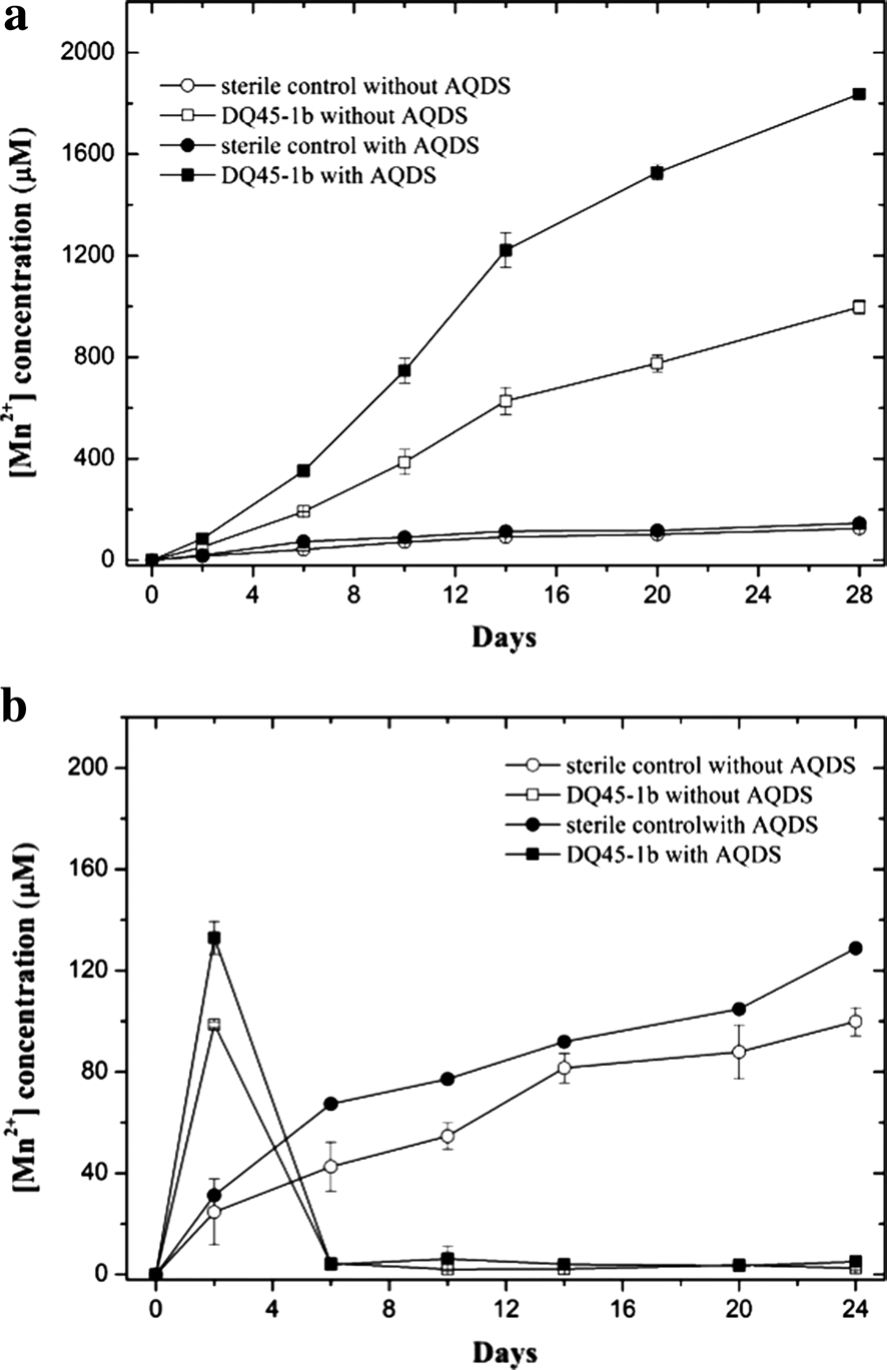
Aqueous Mn^2+^ concentration during birnessite reduction with/without AQDS under anaerobic condition (**a**) and aerobic condition (**b**).

Addition of the humic acid analog AQDS generally enhanced Mn^2+^ release rates under anaerobic condition (Fig. 4a). The Mn^2+^ release rate with AQDS was observed to be approximately one time higher than that without AQDS in the initial 2–14 days and more than 60% higher in 14–28 days (Table 3). Consistently, the AOS of the insolubles decreased from the initial 3.92 to 2.57 with AQDS and to 3.35 without AQDS.

**Table 3:** Microbial Mn reduction with or without AQDS

	AQDS	Mn^2+^ release rate in 2–14 days (μM/day)	Mn^2+^ release rate in 14–28 days (μM/day)	AOS_(total Mn)_	AOS_(insoluble Mn)_	Reduction extent (%)
Anaerobic	−	48.0	26.5	2.90	3.35	53
	+	95.1	43.7	2.17	2.57	91
Aerobic	−	/	/	3.44	3.44	25
	+	/	/	3.80	3.80	6

Samples were biotreated with cell concentration of 2.5 × 10^9^ cell/mL for 28 days under anaerobic conditions and 24 days under aerobic conditions; (+) means with AQDS; (−) means without AQDS; (/) means not detected.

Under aerobic condition, a slight enhancement of Mn^2+^ release rates was observed in the presence of AQDS in the first 2 days (Fig. 4b). However, unlike in anaerobic experiments, the addition of AQDS did not enhance the Mn reduction extent, and the AOS of the insolubles was 3.80 with AQDS and 3.44 without AQDS (Table 3).

**Fig. 5:**
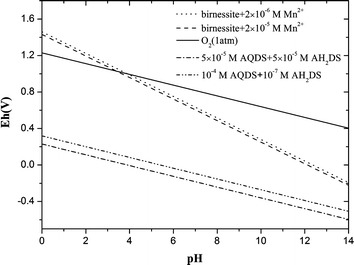
Reduction potential of O_2_, birnessite (at 2 × 10^−5^ and 2 × 10^−6^ M Mn^2+^ activity) [42] and AQDS (at 5 × 10^−5^ M AQDS plus 5 × 10^−5^ M AH_2_DS and 10^−4^ AQDS M plus 10^−7^ M AH_2_DS activity) [43] as a function of pH.

It is believed that AQDS could enhance the rate and extent of microbial metal reduction by shuttling electrons from bacteria to mineral surfaces and thus eliminating the requirement for direct contact of bacteria with electron acceptors [18, 37–40]. The possible reduction of AQDS by cells gave rise to biogenic AH_2_DS (reduced state of AQDS), which then undertook chemical reduction of Mn(IV) [38, 40]. This mechanism could explain the observed increase in both the rate and extent of Mn reduction under anaerobic condition. Under aerobic condition, electrons were transferred to AQDS prior to birnessite and O_2_ [41]. Birnessite and O_2_ competed to accept electrons from biogenic AH_2_DS. At neutral to alkaline environment, the redox potential of O_2_ was higher than that of birnessite, especially at alkaline pH (Fig. 5). So the biogenic AH_2_DS may be preferentially to be oxidized by O_2_. In aerobic bio-treatment, AQDS as an electron shuttle essentially accelerated electron transfer between bacteria and O_2_, which finally lead to the inhibition of Mn(IV) reduction.

### Mineral characterization of bioreduced samples

**Fig. 6:**
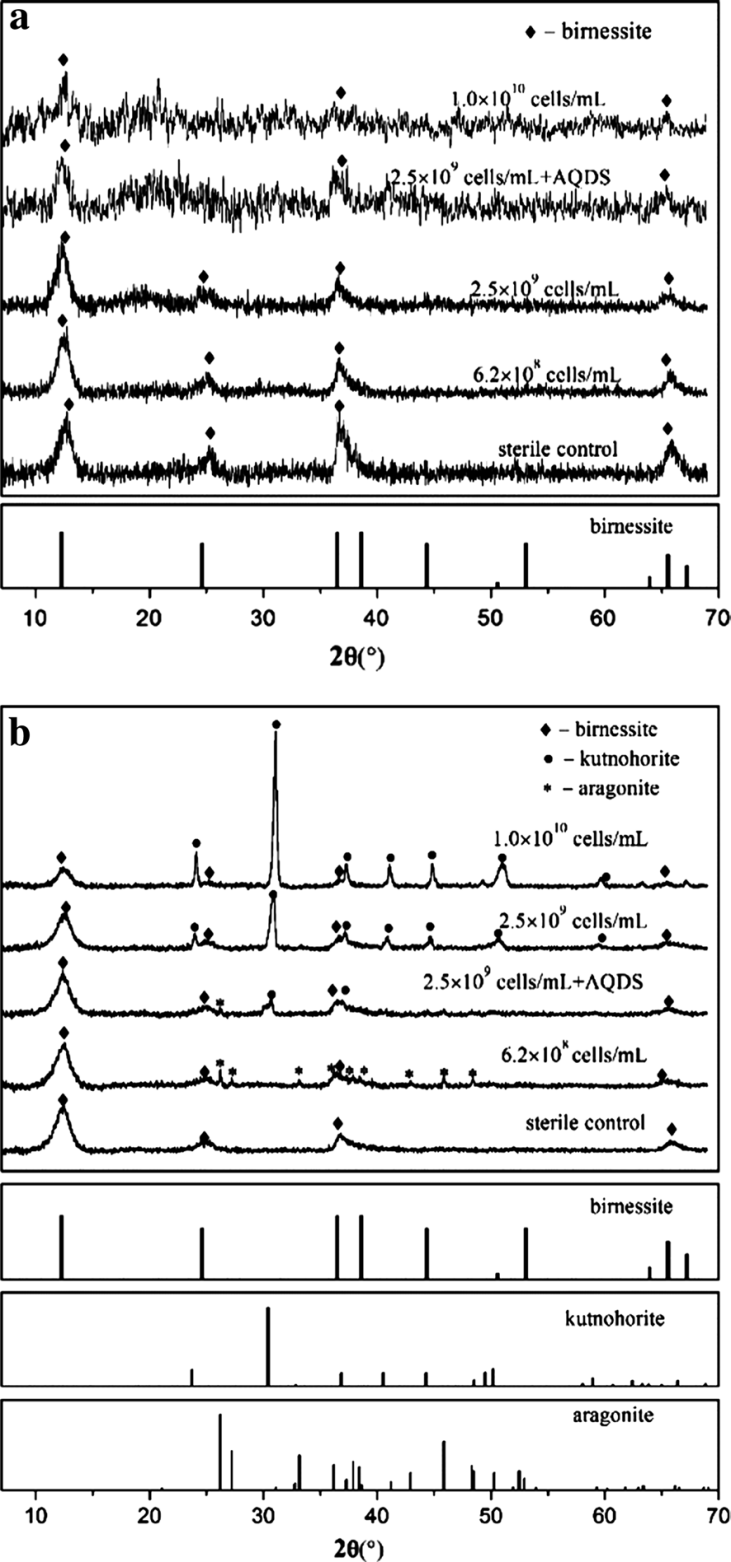
XRD patterns of bioreduced samples under anaerobic (**a**) and aerobic condition (**b**).

Under anaerobic condition, no visible color change in the residual insolubles was observed and the quantity of insoluble in bio-treatments obviously decreased after the experiments. The XRD patterns of residuals in all anaerobic bio-treatments showed the characteristic peaks of birnessite (JCPDS: 23-1239) (Fig. 6a), but with significant loss in peak strength as compared to sterile controls, which was in agreement with the visual observations of bio-induced dissolution of birnessite. No other secondary minerals were detected by XRD.

It was also found that the peak strength decreased as the cell concentration increased, which was attributed to the improved reduction extent of birnessite by higher cell concentration (Fig. 1; Table 1). Besides, the addition of AQDS also significantly accelerated the microbial Mn reduction (Fig. 4a), thereby resulted in more complete dissolution of birnessite and poorer quality XRD patterns of residuals (Fig. 6a).

**Fig. 7:**
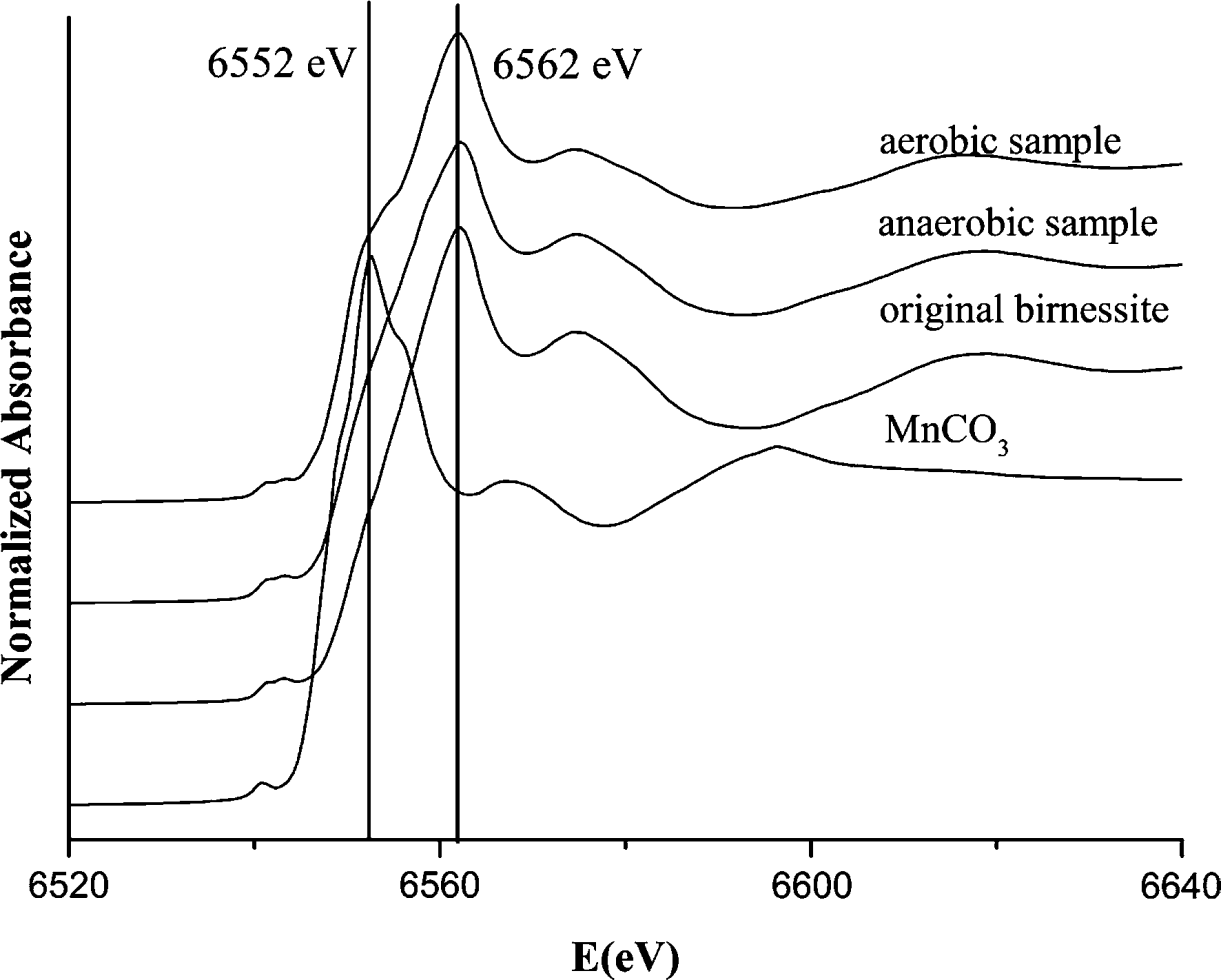
XANES spectra of original birnessite and bioreduced samples in the system with an initial cell concentration of 2.5 × 10^9^ cells/mL.

Further, by comparing the XANES spectra of the residuals in anaerobic bio-treatment to the original birnessite (Fig. 7), we can find a similar peak assigned to Mn(IV) at 6,562 eV and a weak shoulder assigned to Mn(II) at 6,552 eV [44, 45], the latter of which was probably ascribed to adsorbed Mn(II) from birnessite reduction. Taken together, all these data demonstrated the anaerobic reduction of birnessite by DQ12-45-1b released Mn(II) and caused the dissolution of birnessite.

**Fig. 8:**
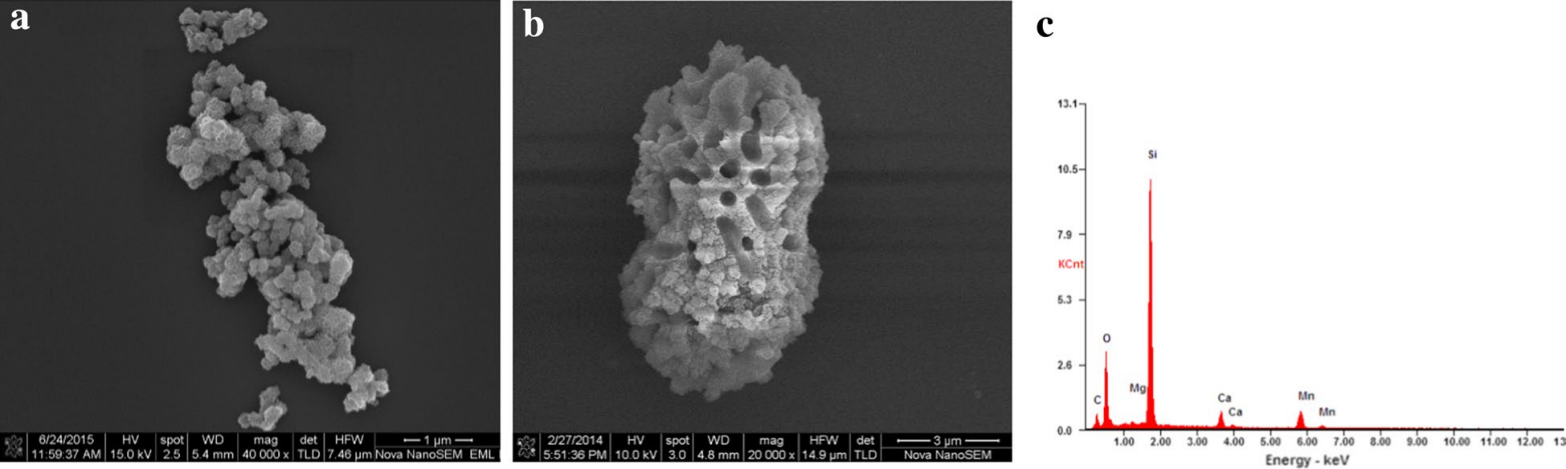
SEM of original and bioreduced samples. **a** SEM of original *globular-flower-like* birnessite; **b** SEM of *spindle-shape* mineral after microbial reduction under aerobic condition; **c** EDS of *spindle-shape* mineral after microbial reduction under aerobic condition.

In aerobic bio-treatments, white suspensions in strong color contrast to black birnessite were observed, indicating the possible precipitation of new mineral phases. Under SEM, some spindle-like aggregations were found and their surface appeared with many holes whose size and shape were very similar to a single cell (Fig. 8b). The EDS results (Fig. 8c) indicated the aggregations mainly consisted of Mg, Ca, Mn, C and O, which was very different from the composition of birnessite. Consistently, XRD patterns of aerobic bioreduced samples showed several new peaks at 2θ = 2.39°, 30.8° and 50.7°, which were assigned to (012), (104) and ($1{\bar{1}}6$) reflections of kutnohorite [Ca(Mn,Mg)(CO_3_)_2_; JCPDS: 084-1290], respectively (Fig. 6b). Thereafter, an obvious shoulder feature at approximately 6,552 eV in the XANES spectra of the residuals in aerobic bio-treatment verified the formation of Mn(II)-bearing minerals.

With the growth of stain DQ12-45-1b, carbon dioxide accumulation from the oxidation of acetate and the increased pH value caused high CO_3_^2−^ activity, therefore leading to the precipitation of carbonate minerals. Accordingly, the quantity of produced carbonates was positively associated with the inoculated cell concentration (Fig. 6b). Surprisingly, only the sample with the lowest cell concentration of 6.2 × 10^8^ cells/mL did not show any peaks of kutnohorite, but showed the existence of aragonite (CaCO_3_; JCPDS: 05-0453) (Fig. 6b). This phenomenon was in agreement with the considerably low concentration of Mn^2+^ (Fig. 3a) and high value of AOS (Table 2) as measured before. Mn(II) produced by bioreduction combined with carbon dioxide produced by acetate metabolism, as well as an alkaline pH environment given by cell growth, finally resulted in the formation of Mn(II)-bearing carbonate (kutnohorite). The concentration of Mn(II) produced in bio-treatment with cell concentration of 6.2 × 10^8^ cells/mL was too low to thermodynamically favor the formation of kutnohorite. And the insufficient supply of Mn(II) resulted in the formation of aragonite instead. Although Mn(II) re-oxidation by O_2_ competed with Mn reduction in aerobic treatments, once the net content of produced Mn(II) reached a proper level, Mn(II) would quickly precipitate and not allow for further oxidation by O_2_. This point could be drawn from the two bio-systems with higher cell concentration. Accordingly, the system with the highest cell concentration of 1.0 × 10^10^ cells/mL gave rise to the most amount of kutnohorite as indicated by XRD results (Fig. 6b). Since DQ12-45-1b preferentially transferred electrons to AQDS and the presence of AQDS facilitated O_2_ reduction but interfered Mn reduction, a relatively smaller proportion of kutnohorite was obtained in the presence of AQDS as compared to that without addition of AQDS (Fig. 6b).

## Conclusion

Birnessite reduction in presence of DQ12-45-1b was observed in both anaerobic and aerobic conditions, and the Mn(IV) reduction proceeded at a more rapid rate when inoculated with higher cell concentration. The extent of Mn(IV) reduction in aerobic conditions was lower than that in anaerobic conditions due to the re-oxidation by oxygen or competition with oxygen respiration. In anaerobic conditions, addition of AQDS improved Mn(IV) reduction extent and accelerated Mn(II) production rate, which ultimately promoted birnessite dissolution. In aerobic treatments, indirect effects ascribed from bacterial metabolism, such as changes of pH, consumption of oxygen and release of metabolites etc., gave a profound influence on the balance between Mn(IV) reduction and Mn(II) re-oxidation, which ultimately lead to the final AOS of Mn oxides, and decided the insoluble products. The presence of AQDS and O_2_ was demonstrated to interfere Mn(IV) reduction and result in low reduction extent of birnessite. The formation of Mn(II)-bearing carbonate (kutnohorite) in aerobic conditions depended on how fast and how far birnessite was reduced to give rise to Mn(II) available for precipitation.

## Additional file

Additional file 1:
**Figure S1.** Acetate concentrations during aerobic birnessite reduction with different initial cell concentrations.
