# Supplementary material for: Aerobic and anaerobic reduction of birnessite by a novel Dietzia strain
Source: Geochem Trans. 2015 Aug 8;16:11. doi: 10.1186/s12932-015-0026-0 (PMC4528715; doi:10.1186/s12932-015-0026-0)
Supplement: Additional file 1: — Figure S1. Acetate concentrations during aerobic birnessite reduction with different initial cell concentrations. [file 12932_2015_26_MOESM1_ESM.docx]

Aerobic and anaerobic reduction of birnessite by a novel *Dietzia* strain

Huiqin Zhang^1§^, Yan Li^1§^, Xin Wang^1^, Anhuai Lu^1*^, Hongrui Ding^1^, Cuiping Zeng^1^, Xiao Wang^1^, Xiaolei Wu^2^, Yong Nie^2^, Changqiu Wang^1^

^1^The Key Laboratory of Orogenic Belts and Crustal Evolution, School of Earth and Space Sciences, Peking University, Beijing 100871, PR China

^2^Department of Energy and Resources Engineering, College of Engineering, Peking University, Beijing 100871, PR China

Email:

Huiqin Zhang^§^: [hqzhang_pku@163.com](mailto:hqzhang_pku@163.com);

Yan Li^§^: [liyan-pku@163.com](mailto:liyan-pku@163.com);

Xin Wang: wx.pkusess@gmail.com;

Anhuai Lu^*^: [ahlu@pku.edu.cn](mailto:ahlu@pku.edu.cn);

Hongrui Ding: dhr_100@163.com;

Cuiping Zeng: [friend13179@126.com](mailto:friend13179@126.com);

Xiao Wang: [782357130@qq.com](mailto:782357130@qq.com);

Xiaolei Wu: [xiaolei_wu@pku.edu.cn](mailto:xiaolei_wu@pku.edu.cn);

Yong Nie: [nieyong@gmail.com](mailto:nieyong@gmail.com);

Changqiu Wang: [cqwang@pku.edu.cn](mailto:cqwang@pku.edu.cn)

^§^ These two authors contributed equally to this work.

^*^ Corresponding Author

**Figure S1 - Acetate concentrations during aerobic birnessite reduction with different initial cell concentrations.**
